# Primary pulmonary hyalinizing clear cell carcinoma harboring *EWSR1::ATF1* fusion: a case report

**DOI:** 10.1186/s44215-026-00280-3

**Published:** 2026-10-05

**Authors:** Takashi Teishikata, Mariko Hashiguchi, Kozue Nakahara, Yusuke Okamoto, Masafumi Hiratsuka, Keiji Kamohara

**Affiliations:** 1 Department of Thoracic and Cardiovascular Surgery, Faculty of Medicine, Saga University, 5-1-1 Nabeshima, Saga, 849-8501 Japan; 2 Division of Pathology, Department of Pathology and Microbiology, Faculty of Medicine, Saga University, 5-1-1 Nabeshima, Saga, 849-8501 Japan

**Keywords:** Hyalinizing clear cell carcinoma, Mucoepidermoid carcinoma, *MAML2*, *EWSR1*, Case report

## Abstract

**Background:**

Hyalinizing clear cell carcinoma (HCCC) is a rare salivary gland–type tumor that occasionally arises in the lung. As it shows substantial morphological overlap with mucoepidermoid carcinoma (MEC), definitive diagnosis can be challenging. Therefore, molecular testing is essential, as MEC typically harbors *CRTC1/3::MAML2* fusions, whereas HCCC is characterized by *EWSR1* fusions.

**Case presentation:**

A 60-year-old woman presented with persistent cough and hemoptysis. Imaging revealed a 3.0-cm solid nodule in the left lower lobe adjacent to the bronchus. She underwent robot-assisted left lower lobectomy with lymph node dissection. Histologically, the tumor consisted of uniform epithelioid cells with clear-to-pale eosinophilic cytoplasm arranged in nests and cords within prominent hyalinized stroma. Cytologic atypia was minimal. Immunohistochemically, tumor cells were positive for CK7, CK5/6, and p40 and negative for TTF-1. Venous invasion was present without lymph node metastasis. Break-apart fluorescence in situ hybridization revealed rearrangements of *EWSR1* and *ATF1*, strongly supporting the presence of an *EWSR1::ATF1* fusion, whereas *MAML2* rearrangement was absent. The patient remains recurrence-free 1 year after surgery.

**Conclusion:**

Accurately distinguishing primary pulmonary HCCC, which is rare and may mimic MEC, is essential for appropriate pathological classification and prognostic assessment. It is also important for thoracic surgeons to consider HCCC during the differential diagnosis of centrally located or endobronchial pulmonary tumors, particularly when a definitive preoperative diagnosis is challenging. Integrating histopathology with molecular testing for *EWSR1* and *MAML2* rearrangements is critical for accurate diagnosis.

## Background

Hyalinizing clear cell carcinoma (HCCC) is a rare salivary gland-type tumor that occasionally arises from the tracheobronchial glands. As HCCC exhibits diverse morphological features, its diagnosis can be challenging. In particular, distinguishing between HCCC and mucoepidermoid carcinoma (MEC), which is another salivary gland-type tumor with overlapping histopathological characteristics, is difficult. Molecular testing is essential because most MECs harbor *CRTC1/3::MAML2* fusions, whereas nearly all HCCC cases show *EWSR1::ATF1* or *EWSR1::CREM* fusions.

Although complete surgical resection remains the key therapeutic strategy for localized HCCC and MEC tumors, accurate classification is critical for appropriate prognostic assessment, postoperative surveillance, and the reliable accumulation of clinicopathological data to better define the biological behavior of such rare tumors.

We report a rare case of primary pulmonary HCCC in which molecular findings strongly supporting an *EWSR1::ATF1* fusion were critical for establishing the final diagnosis.

Written informed consent was obtained from the patient for publication of this case report and any accompanying images.

## Case presentation

A 60-year-old woman presented with a 2-month history of persistent cough and intermittent hemoptysis. Her medical history included type 2 diabetes mellitus. She was a former smoker with a 10-pack-year smoking history. Physical examination results were unremarkable, and routine laboratory test results, including those for serum tumor markers, were within normal limits.

**Fig. 1:**
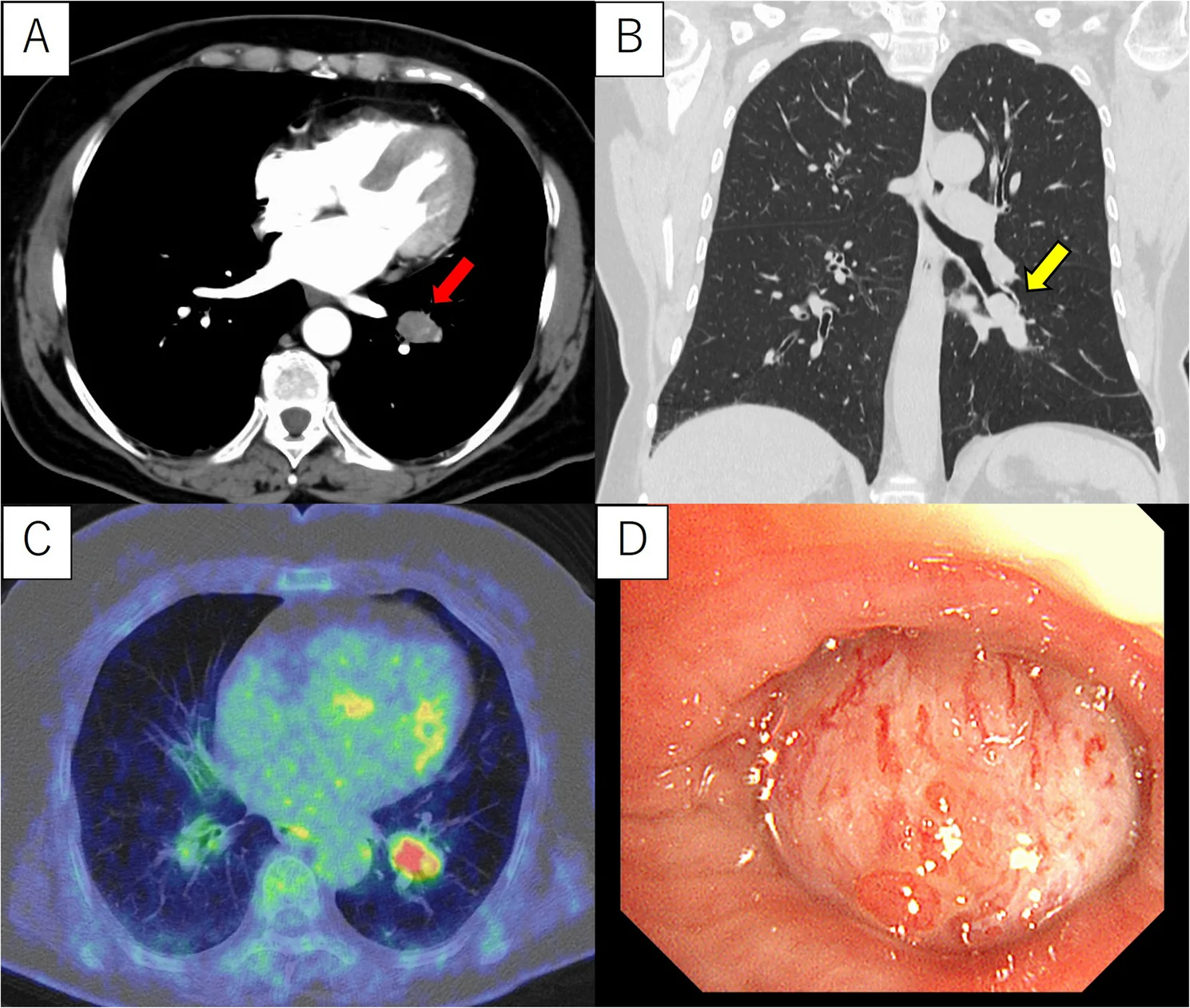
**A**, **B** Chest computed tomography images revealing a well-defined 3.0-cm nodule protruding into the lumen of the left lower lobe bronchus (B9) (red and yellow arrows). **C** Positron emission tomography–computed tomography showing increased fluorodeoxyglucose uptake in the same lesion. **D** Bronchoscopic examination revealing a smooth-surfaced protruding endobronchial mass obstructing the lumen of the left B9 bronchus

Chest computed tomography revealed a well-defined 3.0-cm solid nodule protruding into the lumen of the left lower lobe bronchus (B9) (Fig. 1A, B). Positron emission tomography–computed tomography revealed increased fluorodeoxyglucose uptake in the lesion, with a maximum standardized uptake value (SUVmax) of 8.9, without evidence of abnormal uptake elsewhere (Fig. 1C). Bronchoscopy revealed a smooth-surfaced protruding endobronchial mass arising from the B9 bronchus (Fig. 1D). Endobronchial biopsy was not performed because the lesion appeared to be hypervascular, raising concern regarding procedure-related bleeding.

Thus, a specific histological diagnosis could not be established preoperatively. Radiological and bronchoscopic differential diagnoses included primary pulmonary malignancies, particularly salivary gland-type tumors, such as MEC, as well as carcinoid, metastatic, and benign endobronchial tumors. Given that the patient’s hemoptysis, tumor size, and relatively high FDG uptake (SUV_max_ 8.9) raised concern regarding malignancy, the lesion was clinically treated as a primary lung malignancy and staged as cT1cN0M0 (Stage IA3). We performed robot-assisted left lower lobectomy with systematic mediastinal lymph node dissection for both definitive diagnosis and treatment.

**Fig. 2:**
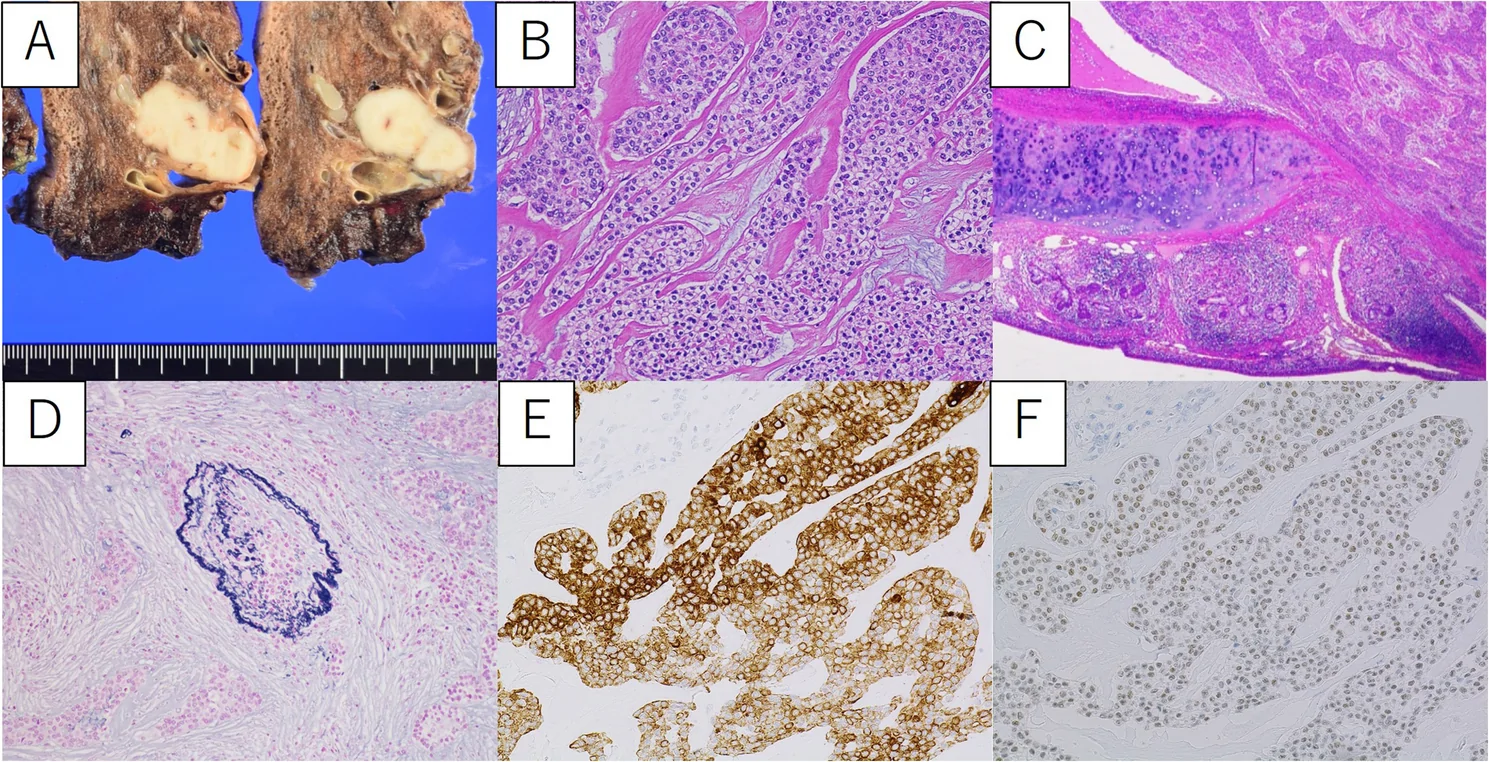
**A** Gross examination of the resected specimen revealing a firm, solitary, well-circumscribed yellowish-white mass measuring 3.0 cm in the greatest dimension, located near the bronchial resection margin. **B**, **C** Histological examination showing tumor cells with clear-to-pale eosinophilic cytoplasm embedded in prominent hyalinized stroma (**B**), and tumor located within the bronchial wall adjacent to the bronchial cartilage, with normal bronchial submucosal glands in close proximity (**C**). (H&E; B, ×200; C, ×20). **D** Weigert elastic fiber staining revealing venous invasion. **E**, **F** Immunohistochemical analysis showing positivity for CK7 (**E**), and most tumor cells demonstrate nuclear positivity for p40 (**F**)

Gross examination of the resected specimen revealed a firm, solitary, well-circumscribed yellowish-white mass measuring 3.0 cm in the greatest dimension, located near the bronchial resection margin at B9 (Fig. 2A). Histological examination of hematoxylin and eosin-stained sections revealed a neoplasm composed of relatively uniform small- to medium-sized epithelioid cells with clear-to-pale eosinophilic cytoplasm and round-to-oval nuclei with inconspicuous nucleoli. Marked nuclear pleomorphism, brisk mitotic activity, and tumor necrosis were absent.

The tumor cells were arranged in cords, nests, and trabeculae embedded in prominent, hyalinized acellular stroma (Fig. 2B). Microscopic examination revealed that the tumor was located within the bronchial wall adjacent to the bronchial cartilage, and normal bronchial submucosal glands were identified in close proximity to the tumor (Fig. 2C). These findings supported a bronchial submucosal gland origin. Tumor infiltration via the bronchial wall into the adjacent peripheral alveolar tissue was also observed. Additionally, venous invasion was noted (Fig. 2D), while lymphatic invasion, perineural invasion, pleural invasion, and lymph node metastases were absent. The bronchial resection margins were tumor free.

Immunohistochemical analysis showed that the tumor cells were positive for CK7 (Fig. 2E) and CK5/6, and most tumor cells demonstrated nuclear positivity for p40 (Fig. 2F). The Ki-67 (MIB-1) labeling index was approximately 1%. The tumor cells tested negative for TTF-1, S100, SOX10, GFAP, synaptophysin, chromogranin A, and smooth muscle actin.

**Fig. 3:**
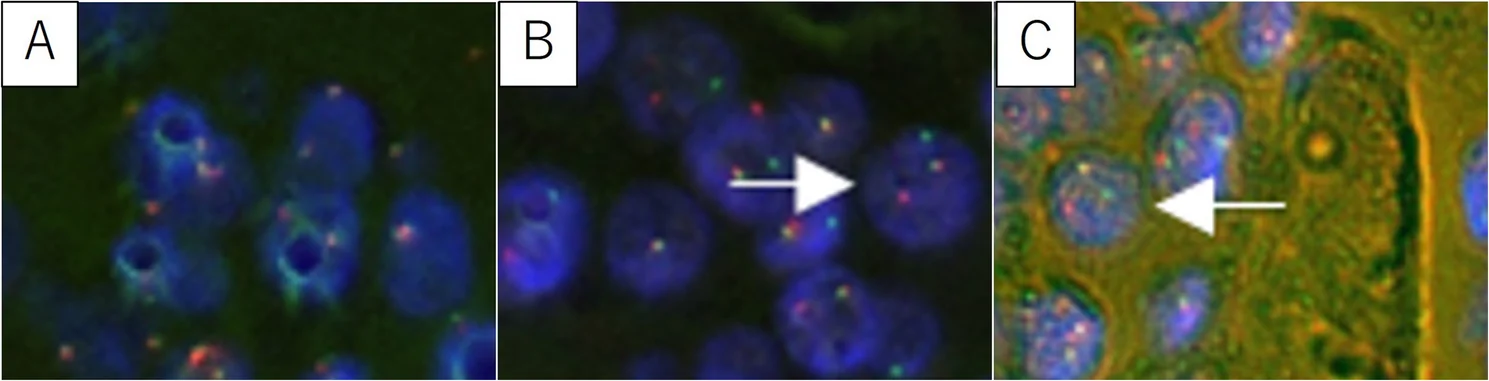
Break-apart fluorescence in situ hybridization analysis showing no evidence of *MAML2* gene rearrangement (**A**). Contrastingly, split signals are observed for *EWSR1* (**B**) and *ATF1* (**C**), strongly supporting the presence of an *EWSR1::ATF1* fusion

Based on histological and immunohistochemical findings, the differential diagnoses included low-grade MEC with clear cell features and HCCC. Given the significant morphological overlap between these entities, additional molecular studies were conducted. FISH analysis showed no evidence of *MAML2* gene rearrangement (Fig. 3A). In contrast, dual-color break-apart FISH demonstrated split signals for *EWSR1* (Fig. 3B) and *ATF1* (Fig. 3C), strongly supporting an *EWSR1::ATF1* fusion. These molecular findings supported the definitive diagnosis of HCCC.

No lymph node metastases were identified, and the final pathological stage was pT1cN0M0 (Stage IA3). The patient was followed up for 1 year after surgery and showed no evidence of recurrence.

## Discussion

HCCC is a distinctive salivary gland-type carcinoma that most commonly arises in the oral cavity and oropharynx and is now recognized as a rare primary tumor of the lung. Primary pulmonary HCCC is presumed to originate from the tracheobronchial submucosal glands and remains exceedingly uncommon. To date, only a limited number of molecularly confirmed cases of primary pulmonary HCCC have been reported, suggesting that this entity remains rare and is likely underrecognized in routine diagnostic practice [1–3].

Histologically, pulmonary HCCC closely resembles its salivary gland counterpart and is characterized by nests, cords, or trabeculae of tumor cells with clear-to-pale eosinophilic cytoplasm embedded in hyalinized or occasionally myxoid stroma [1, 3, 4]. Cytological atypia is generally mild, mitotic activity is low, and tumor necrosis is uncommon, supporting its classification as a low-grade malignancy. In contrast, pulmonary MEC is typically composed of varying proportions of mucous, epidermoid, and intermediate cells. Nevertheless, morphological overlap between HCCC and low-grade MEC is highly probable, particularly when mucin-producing cells in MEC are inconspicuous or absent [5]. Consequently, distinguishing between these two entities based solely on histological findings is often challenging.

Immunohistochemically, pulmonary HCCC frequently expresses squamous markers, such as p40, p63, and CK5/6 but lacks lineage-specific markers that would clearly distinguish it from MEC or other salivary gland-type tumors of the lung [1, 4]. Pulmonary MEC also frequently expresses squamous markers, including p63 and p40 [5]. In addition, markers associated with myoepithelial or neuroendocrine differentiation are typically negative. Owing to this overlapping immunophenotype, immunohistochemistry alone is insufficient for a definitive diagnosis, and pulmonary HCCC may be misclassified as MEC or other clear cell-rich pulmonary neoplasms.

Therefore, molecular analysis is indispensable for accurate diagnosis. MEC is characterized by recurrent *CRTC1::MAML2* or *CRTC3::MAML2* gene fusions, which are regarded as the defining molecular alterations in this tumor type [5, 6]. In contrast, HCCC is characterized by *EWSR1* gene fusions, most commonly involving *ATF1* or *CREM* as fusion partners [3, 4]. In this case, FISH revealed the absence of *MAML2* rearrangement and presence of *EWSR1* and *ATF1* rearrangements. Together with the characteristic histomorphology, these findings strongly supported an *EWSR1::ATF1* fusion and diagnosis of HCCC. This molecular profile is consistent with that of previously reported pulmonary HCCC cases and underscores the critical diagnostic value of molecular testing for salivary gland-type lung tumors with overlapping histological features.

Clinically, pulmonary HCCC should be considered in the differential diagnosis of centrally located or endobronchial tumors. Bronchoscopically, HCCC often appears as a smooth-surfaced or polypoid lesion, whereas squamous cell carcinoma may present as a more irregular or ulcerated lesion; however, adenoid cystic carcinoma may similarly present as a submucosal lesion with intact mucosa [7–9]. Although a definitive diagnosis of HCCC can be established using bronchoscopic biopsy when the representative tissue is obtained, diagnosis based on small biopsy specimens may be challenging because of overlapping morphological and immunohistochemical features [7, 8]. Therefore, molecular testing for *EWSR1* rearrangements or *EWSR1*-associated fusions is particularly useful in such cases. Here, biopsy was avoided because the lesion appeared to be hypervascular, raising concern regarding bleeding.

Although complete surgical resection remains the principal treatment strategy for localized HCCC and MEC, accurately distinguishing between them is clinically relevant. Pulmonary HCCC is generally considered a low-grade malignancy with a favorable prognosis after complete resection. However, its long-term biological behavior remains unclear, owing to the limited number of reported cases. Therefore, the misclassification of HCCC as MEC or another clear cell-rich pulmonary neoplasm may lead to inaccurate prognostic assessment and postoperative surveillance. Moreover, accurate classification is critical to ensure the reliable accumulation of clinicopathological data required to better define the biological behavior and long-term outcomes of this exceptionally rare pulmonary tumor.

Most reported cases of primary pulmonary HCCC follow an indolent course after complete surgical resection. Tumors are often detected incidentally or present with nonspecific respiratory symptoms such as cough or hemoptysis [1–3]. Lymph node metastasis and local recurrence have been documented in a few cases; however, disease-related mortality appears to be exceedingly rare, supporting the generally favorable prognosis for HCCC. Surgical resection with negative margins is the treatment of choice; however, the role of adjuvant therapy has not yet been established. In the present case, the tumor demonstrated a relatively high FDG uptake (SUVmax 8.9) despite low-grade histological features. However, the Ki-67 (MIB-1) labeling index was approximately 1%, mitotic figures were scarce, and tumor necrosis was absent, supporting the low-grade biological behavior of this tumor. Previous reports have similarly described low proliferative activity in pulmonary HCCC, with generally low Ki-67 indices despite variable radiologic findings.

Compared with previously reported cases, this case is notable for venous invasion, a histological feature rarely described in pulmonary HCCC. Its prognostic significance remains unclear because of the limited number of cases and short follow-up periods. Despite this finding, the patient remained free of recurrence at 1 year after surgery. Given the paucity of long-term outcome data, careful surveillance is warranted, particularly in cases with invasive features.

Primary pulmonary HCCC is a rare salivary gland-type carcinoma that should be considered in the differential diagnosis of centrally located or endobronchial pulmonary tumors. When a definitive preoperative diagnosis cannot be safely obtained, complete surgical resection may serve both diagnostic and therapeutic purposes. Integrating histomorphological findings with molecular testing for *EWSR1* and *MAML2* rearrangements is important for accurate diagnosis. Additional molecularly confirmed cases with long-term follow-up are needed to clarify its biological behavior and optimal management.

## Conclusion

We report a rare case of primary pulmonary HCCC harboring an *EWSR1::ATF1* fusion. Despite the relatively high FDG uptake on PET-CT, the tumor demonstrated low proliferative activity with a Ki-67 labeling index of approximately 1%, supporting its low-grade biological behavior. When salivary gland-type tumors with clear cell features are encountered in the lung and histomorphology is inconclusive, molecular testing for *EWSR1* and *MAML2* fusions is essential for an accurate diagnosis.

## Data Availability

The datasets used and/or analysed during the current study are available from the corresponding author on reasonable request.

## Abbreviations

HCCC: Hyalinizing clear cell carcinoma
MEC: Mucoepidermoid carcinoma
FISH: Fluorescence in situ hybridization
